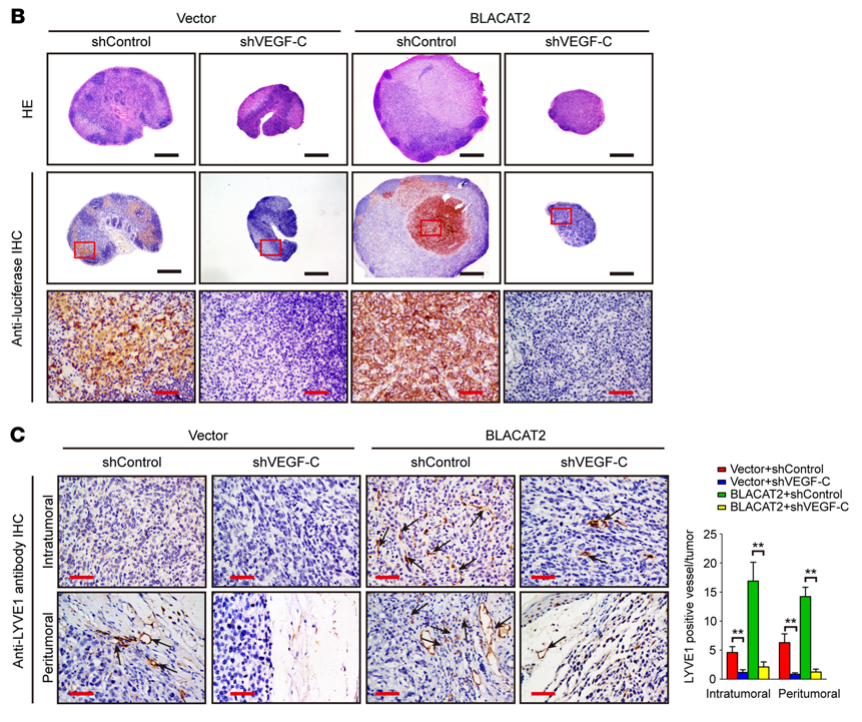# Long noncoding RNA BLACAT2 promotes bladder cancer–associated lymphangiogenesis and lymphatic metastasis

**DOI:** 10.1172/JCI163716

**Published:** 2022-08-15

**Authors:** Wang He, Guangzheng Zhong, Ning Jiang, Bo Wang, Xinxiang Fan, Changhao Chen, Xu Chen, Jian Huang, Tianxin Lin

Original citation: *J Clin Invest*. 2018;128(2):861–875. https://doi.org/10.1172/JCI96218

Citation for this corrigendum: *J Clin Invest*. 2022;132(16):e163716. https://doi.org/10.1172/JCI163716

The authors recently became aware of errors in Figure 9, B and C. In Figure 9B, the area indicated as magnified in the inset box was incorrect for the Vector/shControl/Anti-luciferase IHC image. In addition, Figure 9C contained an incorrect image for the Vector/shControl/Intratumoral sample. The corrected figure panels are shown below.

The authors regret the errors.

## Figures and Tables

**Figure:**